# Imaging of enthesitis by an LED-based photoacoustic system

**DOI:** 10.1117/1.JBO.25.12.126005

**Published:** 2020-12-17

**Authors:** Janggun Jo, Guan Xu, Elena Schiopu, David Chamberland, Girish Gandikota, Xueding Wang

**Affiliations:** a University of Michigan, Department of Biomedical Engineering, Ann Arbor, Michigan, United States; b University of Michigan, Department of Ophthalmology and Visual Sciences, Ann Arbor, Michigan, United States; c University of Michigan Medical School, Division of Rheumatology, Department of Internal Medicine, Ann Arbor, Michigan, United States; d University of Michigan Medical School, Department of Radiology, Ann Arbor, Michigan, United States

**Keywords:** photoacoustic imaging, hyperemia, light-emitting diode, psoriatic arthritis, tendon inflammation

## Abstract

**Significance**: One key pathological characteristic of seronegative spondyloarthropathy (SpA) is inflammation at the insertion of tendons and ligaments into the bone (enthesitis).

**Aim**: We explore the potential of the emerging photoacoustic (PA) imaging in diagnosis of SpA and review its feasibility in detecting SpA-associated Achilles tendon enthesitis.

**Approach**: A light-emitting diode (LED)-based PA and ultrasound combined system was employed. The PA images, both along the long and the short axes of each Achilles tendon insertion region, were acquired at 850-nm wavelength, which is sensitive in depicting increased blood volume (i.e., hyperemia). To assess the hyperemia indicating enthesis inflammation, two parameters were quantified in the imaged tendons, including the average intensity and the density of the color pixels in the pseudo-color PA images. Ten SpA patients, all of which met Assessment of SpA International Society (ASAS) criteria for SpA and were found to have Achilles enthesitis by clinical exam according to a board-certified rheumatologist, were included in the study.

**Results:** The PA and Doppler ultrasound imaging of Achilles enthesitis resulting from these 10 SpA patients were compared to those from 10 healthy volunteers, leading to statistically significant differences (p<0.05) in the applied t-tests.

**Conclusions**: This preliminary clinical study suggests that the LED-based PA imaging holds a promise for sensitive and objective assessment of SpA enthesitis in an outpatient setting of the rheumatology clinic.

## 1 Introduction

Seronegative spondyloarthropathies (SpA) [psoriatic arthritis (PsA), Ankylosing spondylitis (AS), reactive arthritis (ReA), arthritis associated with inflammatory bowel disease (IBD), and undifferentiated spondyloarthritis (uSpA)] are a heterogeneous family of inflammatory arthritides characterized by Assessment of SpondyloArthritis international Society (ASAS) as having inflammatory back pain, presence of enthesitis, dactylitis, and a variety of other extraarticular manifestations. PsA affects about 30% of patients with psoriasis in the United States.^1^^–^^3^ Early SpA treatment is challenged by reduced imaging sensitivity at early stages of pathology conditions, where symptoms are nonspecific and emerging after radiographic progression occurs, and the average time from onset of symptoms to diagnosis can be as long as 10 years.^4^ The lack of early diagnosis of SpA is due to limited pathognomonic clinical technology in specific imaging of the biomarkers of the disease. Although initially considered as a variant of rheumatoid arthritis (RA), SpA was later defined as a distinct clinical entity.^3^^,^^5^ Clinical assessment of physiological changes associated with SpA is also a problem. Unlike RA-related synovitis, enthesitis is more challenging to detect with ultrasound (US) imaging. Magnetic resonance imaging (MRI) remains an excellent choice for imaging enthesitis, but is costly and not easily accessible. Emerging clinical research on SpA has been focused on studies that identify or validate biomarkers that are sensitive to early diagnosis and disease progression and early responses to therapy.

The physiological symptoms such as hyperemia and hypoxia in soft tissue are directly associated with the pathological condition of the joints, tendons, and entheses affected by inflammatory arthritis. By monitoring these features using the emerging photoacoustic (PA) imaging technology, joint inflammation has been detected and evaluated in previous studies.^6^^–^^13^ Based on highly sensitive optical contrast, PA imaging offers excellent sensitivity in assessing hyperemia,^11^^,^^12^^,^^14^ which is an essential step in the inflammatory cascade and a result of chronic inflammation causing hypervascularization associated with an increased capillary network and distended veins. In addition, by performing dual-wavelength PA imaging, the hypoxia in arthritic joints as another important physiological symptom can be quantitatively measured.^11^ These previous studies on inflammatory joints have suggested that PA imaging, as an add-on to clinical US imaging, could offer additional and valuable diagnostic information for an earlier and more comprehensive assessment of inflammatory arthritis and responses to therapy.

Early symptoms of SpA are nonspecific and could be followed by a period of undifferentiated inflammatory arthritis that is difficult to distinguish from RA. Several distinct clinical features, such as hyperemia in tendons and entheses, distinguish SpA from other forms of inflammatory arthritis. In this initial clinical study on SpA patients, we, for the first time to the best of our knowledge, explored the feasibility of PA imaging in detecting inflammation in human Achilles entheses in patients who met the criteria for ASAS-defined SpA. In this study, a commercially available light-emitting diode (LED)-based PA imaging system was employed. A good performance of this system in detecting inflammation in arthritic joints has been reported.^12^^,^^13^ Using low-cost, small-size, portable, and clinically safe light sources such as LEDs as the illumination source, PA imaging has many advantages. It may accelerate the clinical translation of this emerging medical imaging technology by providing a safe, sensitive, easily adaptable, and clinically useful tool aiding early SpA diagnosis. Imaging results from the SpA patients were compared to those from the healthy volunteers, and the presence of inflammation was confirmed by PA and the routine musculoskeletal US Doppler imaging.

## 2 Methods

**Fig. 1:**
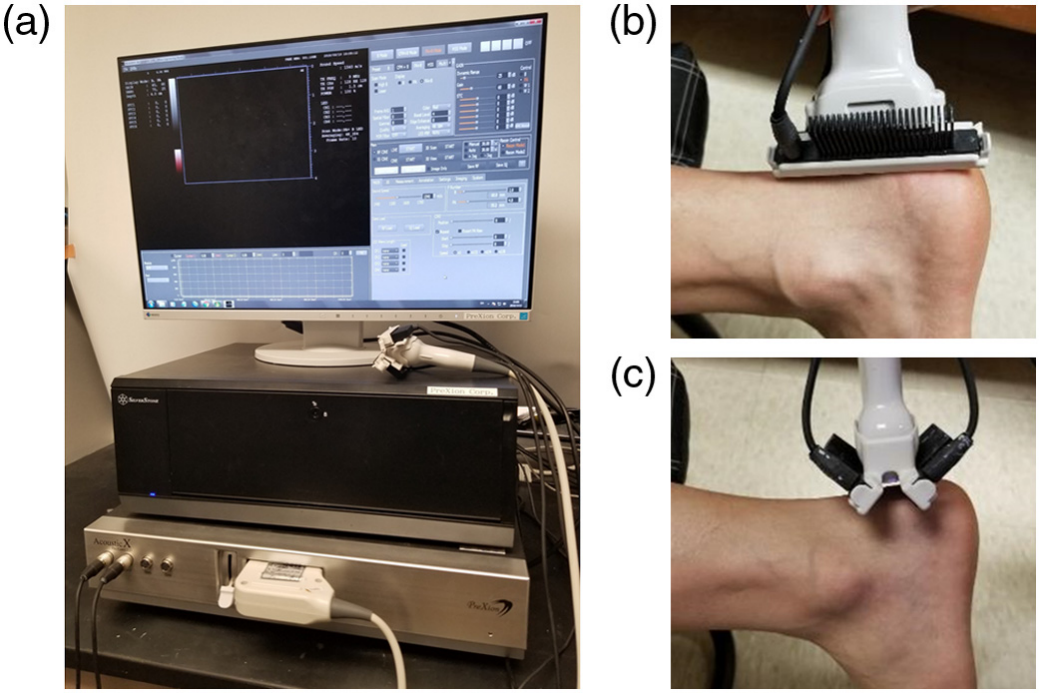
LED-based PA imaging of tendon inflammation. (a) Photograph of the imaging system. (b) Patient’s Achilles tendon scanned with the imaging probe along the long axis of the tendon. (c) Patient’s Achilles tendon scanned with the imaging probe along the short axis of the tendon.

The LED PA imaging system used in this study was built by Cyberdyne (AcousticX, Cyberdyne, Tokyo, Japan), as shown in Fig. 1. The details of this system, including the safety for applications on human subjects, have been introduced in our previous publications.^12^^,^^13^^,^^15^^,^^16^ The system has two LED array bars located on both sides of a US transducer probe with an angle of 45 deg to the center plane of the probe (Fig. 1). The light source has a repetition rate of 4 kHz and a pulse duration of 35 ns. Each pulse provides light energy of 400  μJ at an 850-nm wavelength. The PA signals were acquired at each LED pulse. After processing the PA signals using a frequency bandpass filter, log compression, and time gain compensation, the PA images were reconstructed using the delay-and-sum method and then displayed in pseudocolor. The reconstructed PA image in pseudocolor was processed by the integrated software in AcousticX and displayed on the background gray scale US image after applying dynamic range control to enhance high intensity signals. A consistent gain of 56 was employed during PA imaging of all human tendons in this study. Using the same level of gain, we can compare the PA image intensities between the patient and control groups. To enhance the signal-to-noise ratio, the PA images were averaged over 384 light pulses, leading to an imaging frame rate of ∼10  Hz. The US images acquired using the same probe were displayed in a gray scale, also in real-time. When working with the 7-MHz linear probe with 128 elements, as employed in this study, this system offers a spatial resolution of 310-μm lateral and 250-μm axial, and an image depth up to 30 mm for PA imaging.^12^^,^^13^

In this study, conventional US Doppler imaging was used as the gold standard for confirming the hyperemia in the affected enthuses.^17^^,^^18^ Because the AcousticX imaging system does not provide the Doppler mode, the US Doppler images were acquired by a commercial US unit (Z.ONE PRO, Mindray, Mahwah, New Jersey) working with a linear probe (L14–5W, Mindray). The US Doppler images were acquired at a pulse repetition frequency of 1500 Hz and a scale of +7.5 to −7.5  cm/s to show hyperemia in the enthesis area both along and across the enthesis orientation. The same approximate imaging planes were revisited by the LED-based PA imaging system. The patients’ entheses that did not show prominent flow in the US Doppler imaging were also scanned by the LED PA imaging in search of increased vascularity. The total scanning time, including both Doppler US and LED PA imaging of each human subject, was less than 10 min.

All procedures for human subjects in this study were approved by the Institutional Review Board (IRB) of the University of Michigan Medical School. We performed the study in accordance with the approved protocol. Ten SpA patients, who are either men or women and are over 18 years old, were recruited through the University of Michigan Rheumatology clinics. Board-certified rheumatologists at the University of Michigan Medical School confirmed both the pathological condition of SpA, according to ASAS, and enthesitis. The normal control group includes 10 healthy volunteers who did not have symptoms or signs of enthesitis and no clinical record of any inflammatory arthritis. All participants provided written informed consent.

## 3 Results

**Fig. 2:**
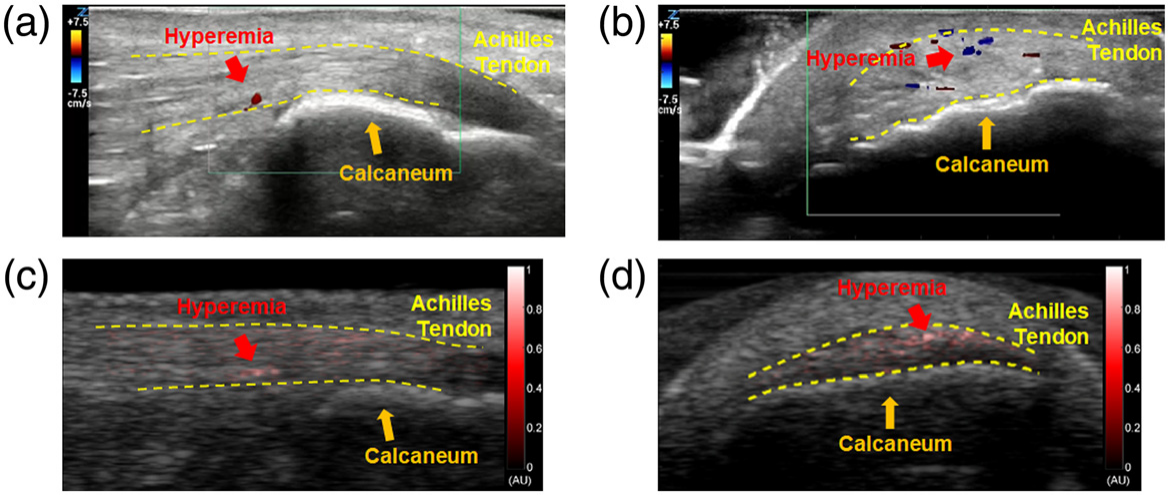
US Doppler and PA imaging of an Achilles tendon (margins outlined by dotted line) of an SpA patient. (a) and (b) US Doppler images of the patient’s Achilles tendon when scanned along the long and the short axes of the tendon, respectively. (c) and (d) PA images of the patient’s Achilles tendon when scanned along the long and the short axes of the tendon, respectively. The pseudocolor PA images were superimposed on the gray-scale B-mode US images.

Figure 2 shows the representative imaging results from an SpA patient. In gray-scale B-mode US images, the areas of distal Achilles tendon that connect the heel bone (Calcaneum) with the calf muscles can be visualized, as marked by the dashed yellow lines. In pseudocolor PA images both along the long and the short axes of the entheses, strong signals reflecting the hyperemia can be clearly recognized. The enthesis inflammation detected by PA imaging was confirmed by the Doppler US imaging, which also showed active Doppler signals both along and across the entheses, although the Doppler signals along the entheses were not as strong.

**Fig. 3:**
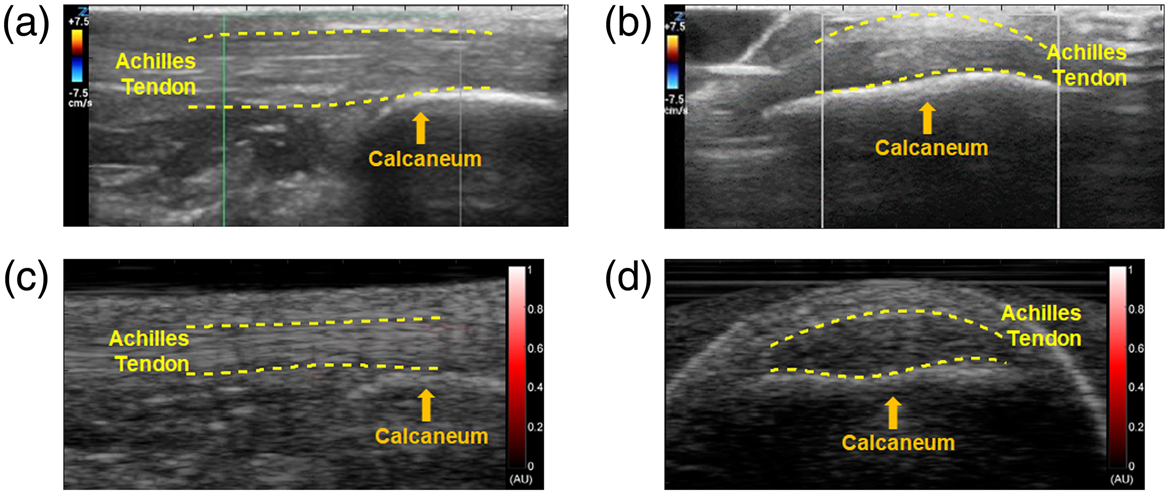
US Doppler and PA imaging of an Achilles tendon of a normal volunteer. (a) and (b) US Doppler images of the volunteer’s Achilles tendon when scanned along the long and the short axes of the tendon, respectively. (c) and (d) PA images of the volunteer’s Achilles tendon when scanned along the long and the short axes of the tendon, respectively. No hyperemia noted.

Figure 3 shows the representative imaging results from a healthy volunteer. Similar to Fig. 2, the enthesis areas can be marked in B-mode US images. However, neither along the long axis of the enthesis nor across the enthesis can the PA images detect any obvious hyperemia signals, indicating there was no enthesis inflammation. This finding was confirmed by the Doppler US imaging, which did not show any activation either along or across the enthesis.

**Fig. 4:**
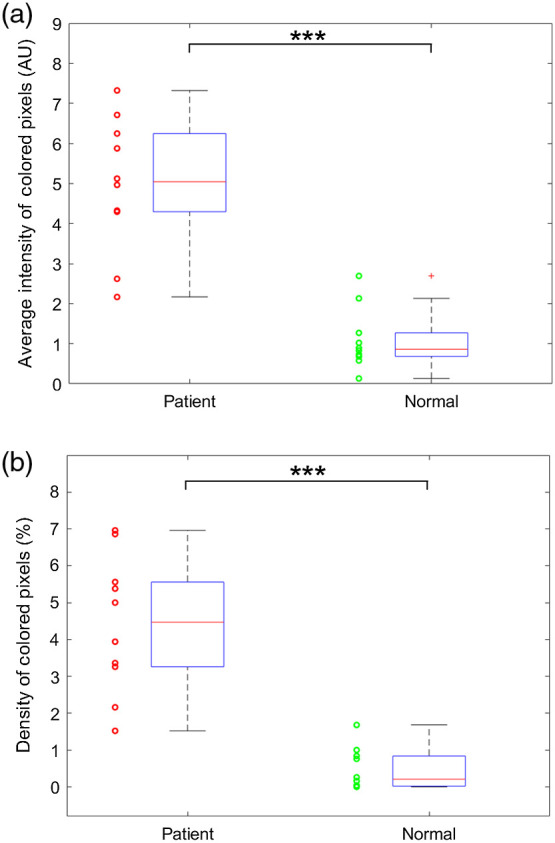
Statistical analyses of the quantified PA imaging results reflecting the detected hyperemia along with the entheses. The results from the group of 10 SpA patients are compared to the results from the group of 10 normal volunteers. (a) The average intensities of the colored pixels in the pseudocolor PA images scanned along the tendons from the two groups. (b) The density of the colored pixels in pseudocolor PA images scanned along the tendons from the two groups. *** indicates p<0.001.

**Fig. 5:**
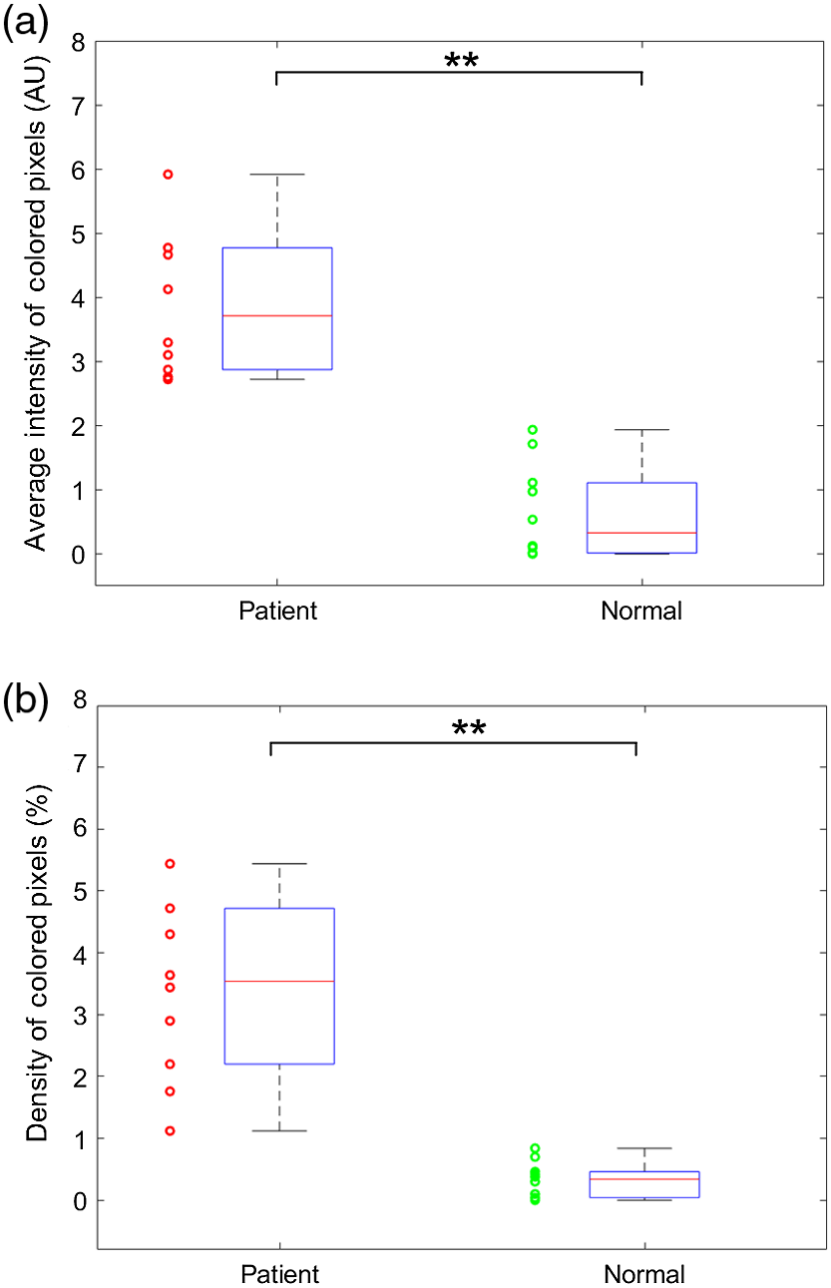
Statistical analyses of the quantified PA imaging results reflecting the detected hyperemia across with the entheses. The results from the group of 10 SpA patients are compared to the results from the group of 10 normal volunteers. (a) The average intensities of the colored pixels in the pseudocolor PA images scanned acrossed the tendons from the two groups. (b) The density of the colored pixels in pseudocolor PA images scanned acrossed the tendons from the two groups. ** indicates p<0.005.

To evaluate the performance of PA imaging for detecting enthesis inflammation, the results from 10 SpA patients and 10 healthy volunteers were statistically compared. With the PA images acquired from each subject, two parameters that objectively reflect the severity of hyperemia were quantitatively analyzed, following the methods in previous publications.^11^^,^^12^ One parameter calculates the average intensity of all the colored pixels within the enthesis area in the PA image; the other parameter calculates the density of the colored pixels in the enthesis area, which is the ratio between the number of colored pixels and the number of all pixels of the tendon area. These two quantified PA parameters from SpA patients and healthy volunteers are compared in Figs. 4 and 5, where the results acquired along the entheses and across the entheses are studied individually. Figure 4(a) shows the box plots of the average intensities of colored pixels in the PA images along the entheses from the two groups, where the mean from the patient group is 4.53 times stronger than that from the healthy volunteer group. Figure 4(b) shows the box plots of the density of colored pixels in the PA images along the entheses from the two groups, where the mean from the patient is 11 times stronger than that from the healthy volunteer group. PA imaging results across the entheses, as shown in Figs. 5(a) and 5(b), demonstrate similar findings. The quantified average intensities of the colored pixels and the densities of the colored pixels from the SpA patient group have 7.5 fold and 13.75 fold enhancements, respectively, compared to the healthy volunteer group. To examine statistically significant differences between the two groups for each of the quantified parameters, a two tailed *t*-test was conducted using the built-in functions of MATLAB^®^ (R2018b, Mathworks, Natick, Massachusetts). The calculated p values were $3.13\times 10^{-6}$ and $5.29\times 10^{-6}$, respectively, for the results of PA images along with the entheses in Figs. 4(a) and 4(b). The p values from PA imaging results across the entheses were $1.66\times 10^{-3}$ and $4.26\times 10^{-3}$ in Figs. 5(a) and 5(b). The statistical results of the t-tests indicate that each quantified parameter can successfully differentiate the SpA patient group and the healthy volunteer group.

**Fig. 6:**
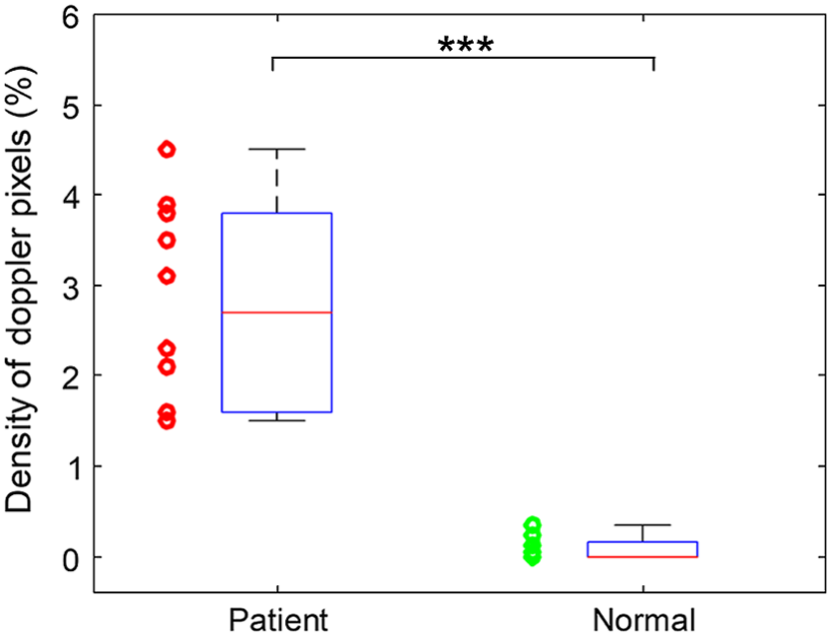
Density of the colored pixels in the US Doppler images scanned along the tendons from the two groups including 10 SpA patients and 10 normal volunteers, respectively. *** indicates p<0.001.

Statistical analysis was also conducted to compare the Doppler US images from the 10 SpA patients and the 10 healthy volunteers. The results in Fig. 6 quantified the percentages of the colored pixels that present Doppler signals in the tendon area. The t-test comparing the Doppler US images from the two subject groups led to a p value of $1.55\times 10^{-6}$.

## 4 Conclusion and Discussion

Enthesitis is part of the ASAS criteria for SpA and can help distinguish this distinct pathological entity from RA;^19^^,^^20^ in addition, it could be an important biomarker in assessing treatment response or disease remission. Past modalities for evaluation of the presence/absence and severity of enthesitis include, but are not limited to, clinical exam, conventional radiography, US, and MRI. Each of these techniques has pros and cons associated with them. For example, clinical assessment of enthesitis has been organized as outcome measures (Maastricht Ankylosing Spondylitis Enthesis Score, Spondyloarthritis Research Consortium of Canada, and Leeds Enthesitis Index) but have never been validated to be used in SpA and have limitations in term of reliability, validity, and sensitivity.^21^ In addition, many of the entheseal points are around areas of accepted tender points for fibromyalgia (prevalent in SpA), which leads to the possibility of misclassification. Conventional radiography detects later stage sequelae of enthesitis such as large erosions, but is not able to detect or quantify smaller erosions or earl soft tissue changes. MRI is a solid modality to evaluate enthesitis, but is expensive and not easily accessible. US is widely used because it is inexpensive, accessible, nonionizing, noninvasive, produces real-time images, and can document both bony abnormalities such as erosions along with soft tissue inflammatory characteristics. US, however, is highly operator dependent.^22^ PA imaging is a potentially excellent imaging modality for enthesitis as it offers excellent sensitivity in assessing hyperemia and can also simultaneously take advantage of all of US’s imaging capabilities.

In this work, via an initial clinical study on SpA patients and healthy volunteers, the emerging PA imaging technique’s clinical potential for the detection of enthesis inflammation was explored. The PA images reflecting hyperemia were acquired using a PA and US combined system where the PA light source is based on two LED arrays. Due to its unique advantages over conventional laser-based PA imaging systems, such as portability, the LED-based system can be more beneficial as a point of care device for screening in a large population.

The results from this initial clinical study demonstrated that the PA images acquired at an 850-nm wavelength shows high sensitivity to enthesis inflammation and can easily detect the spatially distributed hyperemia both along the long axis and the short axis of the affected entheses. Despite the low energy of the light pulse (400  μJ) utilized, the imaging depth enabled by this LED PA imaging system is sufficient to cover the entire Achilles tendon or other superficial tendons. In the statistical results, PA imaging has demonstrated statistical significance to differentiate patients and healthy control subjects with low p values <0.005. In a previous study,^12^ a comparison of PA and US Doppler for imaging of inflammatory arthritis was conducted, which demonstrated that, by leveraging the highly sensitive optical contrast, the PA imaging can achieve better sensitivity in detecting mild inflammation in human peripheral joints.

This clinical study has some limitations. The tendon insertion images acquired in these studies are all two-dimensional (2D). Although acquiring 2D images by the handheld probe is convenient and fits into the current clinical protocol, 2D images along arbitrary imaging planes make the imaging results highly dependent on the operators and difficult for quantitative assessment. Hence, our plan includes developing a three-dimensional (3D) imaging of tendon insertion inflammation, which could present volumetric information for a more accurate evaluation of hyperemia. Another limitation of the current LED technology is that the PA imaging system used in this study has limited optical wavelength options. As a result, all the images were acquired at a single wavelength of 850 nm. Our plan includes PA imaging at two or more wavelengths, which could enable quantitative mapping of hypoxia (i.e., decreased blood oxygen saturation level) as another essential functional hallmark of tendon inflammation. This could be achieved by utilizing either dual-color LED arrays or other light sources working at multiple wavelengths. Despite these limitations, this initial clinical study has successfully demonstrated that PA imaging, as a safe and effective functional imaging modality, could offer an additional practical point of care tool for clinical diagnosis and treatment evaluation of SpA. This study extended the imaging ability for angiogenic microvessels to human tendon enthesitis. Encouraged by the promising results of the PA hyperemia imaging, we will move on to an advanced imaging system performing PA imaging together with US Doppler in a future study. Such a system that can obtain additional Doppler images simultaneously with PA imaging in tendon enthesitis will enable a more accurate comparison between the modalities.
