# Imaging of enthesitis by an LED-based photoacoustic system (Erratum)

**DOI:** 10.1117/1.JBO.26.5.059801

**Published:** 2021-05-17

**Authors:** Janggun Jo, Guan Xu, Elena Schiopu, David Chamberland, Girish Gandikota, Xueding Wang

**Affiliations:** a University of Michigan, Department of Biomedical Engineering, Ann Arbor, Michigan, United States; b University of Michigan Medical School, Department of Ophthalmology and Visual Sciences, Ann Arbor, Michigan, United States; c University of Michigan Medical School, Division of Rheumatology, Department of Internal Medicine, Ann Arbor, Michigan, United States; d University of Michigan Medical School, Department of Radiology, Ann Arbor, Michigan, United States

## Abstract

An error in the first author’s name is corrected.

This article [*J. Biomed. Opt.* **25**(12), 126005 (2020) doi: 10.1117/1.JBO.25.12.126005 was originally published on 17 December 2020 with an error in the name of the first author. Janggun Jo was misspelled as “Junggun Jo.” The error was corrected on 7 May 2021.

